# *De novo* genome assembly of the endemic Italian springtail *Orchesella dallaii* (Collembola: Orchesellidae)

**DOI:** 10.1093/g3journal/jkaf240

**Published:** 2025-10-07

**Authors:** Claudio Cucini, Francesco Nardi, Rebecca Funari, Riccardo Bulletti, Antonio Carapelli, Pietro Paolo Fanciulli, Francesco Frati

**Affiliations:** Department of Life Sciences, University of Siena, Via Aldo Moro, 2, Siena 53100, Italy; National Biodiversity Future Center (NBFC), Piazza Marina, 61, Palermo 90133, Italy; Department of Life Sciences, University of Siena, Via Aldo Moro, 2, Siena 53100, Italy; National Biodiversity Future Center (NBFC), Piazza Marina, 61, Palermo 90133, Italy; Department of Life Sciences, University of Siena, Via Aldo Moro, 2, Siena 53100, Italy; Department of Life Sciences, University of Siena, Via Aldo Moro, 2, Siena 53100, Italy; Department of Life Sciences, University of Siena, Via Aldo Moro, 2, Siena 53100, Italy; National Biodiversity Future Center (NBFC), Piazza Marina, 61, Palermo 90133, Italy; Department of Life Sciences, University of Siena, Via Aldo Moro, 2, Siena 53100, Italy; Department of Life Sciences, University of Siena, Via Aldo Moro, 2, Siena 53100, Italy; National Biodiversity Future Center (NBFC), Piazza Marina, 61, Palermo 90133, Italy

**Keywords:** whole-genome sequencing, mitogenome, biodiversity, conservation, genome assembly, Collembola, Orchesella dallaii

## Abstract

Springtails (Collembola) constitute one of the most diverse and ecologically important groups of basal hexapods, yet remain significantly underrepresented in genomic databases. In this study, we present the first genome assembly of *Orchesella dallaii*, an Italian endemic species, representing a crucial advancement in expanding genomic resources for Collembola. Utilizing PacBio HiFi sequencing combined with an ultra-low input library preparation, we generated a highly contiguous genome assembly (223 contigs covering 304 Mb) that ranks among the most complete ones within the group. Genome annotation, supported by short-read RNA-seq data, predicted 31,769 genes with high completeness. The analysis of repeated sequences revealed a comparatively low abundance of annotated transposable elements. Additionally, the entire mitochondrial genome was assembled and annotated, confirming the gene order characteristic of the Entomobryomorpha lineage. This genomic resource provides a valuable reference for an often-overlooked taxonomic group and offers a basis for future research in comparative genomics, species delimitation, and conservation genetics of Mediterranean soil ecosystems.

## Introduction

Among basal hexapods, Collembola (springtails) represent one of the most diverse and widespread groups (Bellinger et al. 2024). They typically inhabit soil and litter layers across nearly every ecosystem worldwide, from tropical to polar regions, where they play a crucial role in soil food webs (Potapov et al. 2020). Remarkably, their diversification dates back to the Silurian period, approximately 430 million years ago—coinciding with the initial formation of soil layers on land—emphasizing their long-standing and fundamental role in shaping terrestrial ecosystems (Leo et al. 2019). Collembola comprises approximately 9,600 described species (Bellinger et al. 2024). However, this diversity is widely considered underestimated due to the prevalence of cryptic species that remain undetected using traditional methods (e.g. Carapelli et al. 2020; Valle et al. 2025). Consequently, it has been proposed that the true number of Collembola species could range from 65,000 to as many as 500,000 (Cicconardi et al. 2013; Turnbull and Stebaeva 2019).

Beyond their ecological and evolutionary significance, Collembola have also attracted attention in comparative genetics for the presence of β-lactam biosynthesis genes. First described in *Folsomia candida*, these genes include a functional isopenicillin N synthase (IPNS), acquired through horizontal gene transfer (HGT), that enables the production of β-lactam antibiotics (Roelofs et al. 2013). Follow-up studies further revealed a larger gene cluster comprising IPNS, δ-(L-α-aminoadipoyl)-L-cysteinyl-D-valine synthetase (ACVS), and cephamycin genes, transcribed and induced under stress conditions, as well as the presence of β-lactam compounds detected *in vivo* (Suring et al. 2017). Comparative surveys showed that different combinations of these genes are present in different springtail families, suggesting that β-lactam biosynthesis may represent a widespread adaptation to a soil-dwelling lifestyle. However, these orthology analyses were based on early genome and transcriptome data, often incomplete or fragmented, and did not fully capture the entire biosynthetic pathway. High-quality genomic resources are therefore essential to reassess the prevalence, organization, and evolutionary origin of β-lactam genes in Collembola.

In recent years, with the advent of next-generation sequencing technologies, numerous *omics* datasets for Collembola have been generated, ranging from transcriptomics (Faddeeva et al. 2015; Cucini et al. 2021a) to metabarcoding (Leo et al. 2021) and metagenomics (Collins et al. 2023). More recently, there has been growing emphasis on mitogenomic and whole genome data, primarily aimed at resolving the systematics of this complex and still debated group, where multiple evolutionary scenarios have been proposed (i.e. Nardi et al. 2020; Cucini et al. 2021b; Godeiro et al. 2023; Yu et al. 2024). Genomic data have also been employed for species delimitation (Timmermans 2025), ecological genetics (Luan et al. 2022; Bakker et al. 2023), gene family evolution (Faddeeva-Vakhrusheva et al. 2016), and chromosome evolution (Jin et al. 2024). In addition, valuable insights on springtail genomics have been obtained by Schneider et al. (2021) and the Darwin Tree of Life Project (Jaron et al. 2023, 2024; McCulloch et al. 2025).

**Fig. 1.**
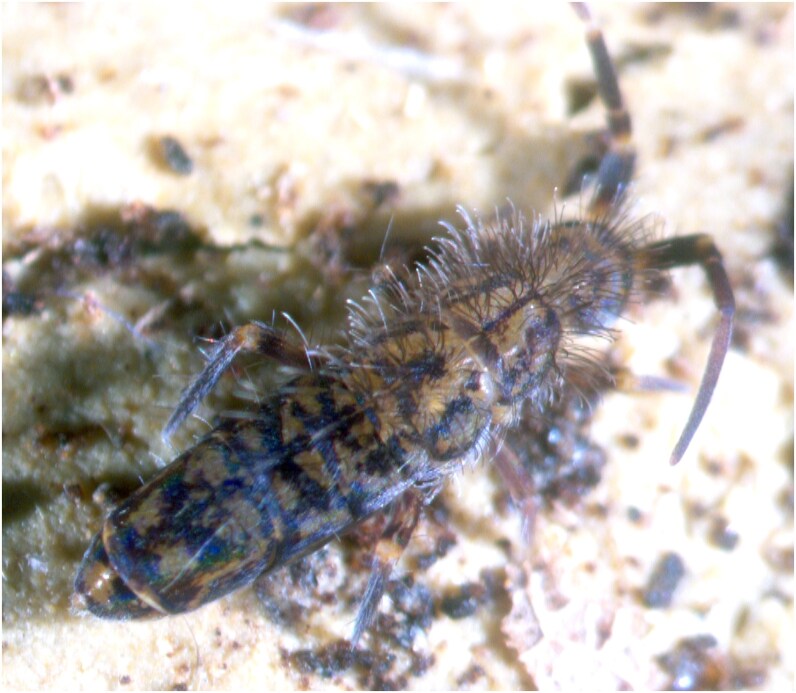
Individual of *Orchesella dallaii* photographed under a stereomicroscope. Adult individuals measure approximately 6 mm in length.

Despite the recent growth in genomic resources, the overall genomic representation of Collembola remains markedly limited. As of April 2025, only 134 reference genomes are deposited in the NCBI Genomes database for Collembola—a notably low number given the estimated diversity of the group. Furthermore, none of these genomes correspond to endemic Italian species. To address this shortfall, we present here the assembly and annotation of the first draft genome of an endemic Italian springtail, *Orchesella dallaii* (Fig. 1), a hemiedaphic species that inhabits the litter layers. This resource lays a critical foundation for future research in conservation genomics for this species and comparative genomics within the group.

## Methods

### Sample collection

Individuals of *O. dallaii* were collected in the wild at Colfiorito (Foligno, Perugia, Italy; coordinates: 43°0′40″ N, 12°51′54″ E), the species’ topotypic locality, in May 2023. Taxonomic identification was conducted based on the morphological description provided by Frati and Szeptycki (1990). Because sex-specific diagnostic features are not described for this species, the sex of the analyzed specimens remains unknown.

### Nucleic acids extraction and sequencing

High-molecular-weight genomic DNA was extracted from a single individual of *O. dallaii* using the Wizard Genomic DNA Purification kit (Promega) following the manufacturer's instructions. Library preparation was performed using the Ultra-Low DNA input workflow for SMRT Sequencing (PacBio), and sequencing was carried out on a Sequel II platform at the Centre for Genomic Research (Liverpool, UK).

Total RNA was in turn extracted from a pool of 3 individuals using the QIAGEN RNeasy Micro kit, following the manufacturer's protocol with modifications as described by Cucini et al. (2024), to enhance transcript diversity. Enrichment for polyadenylated RNAs was performed using the NEBNext poly(A) mRNA Magnetic Isolation Module (New England Biolabs). Library preparation was subsequently carried out using the NEBNext Ultra Directional RNA library Prep Kit for Illumina. Sequencing was performed on an Illumina NovaSeq 6000 platform with a 150 bp paired-end (PE) layout at the Centre for Genomic Research (Liverpool, UK).

### Mitochondrial genome assembly

Reads of mitochondrial origin were extracted from the whole-genome sequencing data of *O. dallaii* and used for mitochondrial genome assembly with MitoHiFi v3.2.1 (Uliano-Silva et al. 2023) prior to nuclear genome assembly. This pipeline identifies organellar reads based on sequence similarity to reference mitochondrial genomes from closely related species. For this purpose, we employed *Orchesella cincta* (NC_032283.1) as a reference. MitoHiFi assembled and circularized the mitochondrial contig, which was subsequently annotated using MITOS (Brent et al. 2013). The annotation was manually curated to improve gene boundary accuracy and was visualized using EZmap (Cucini et al. 2021c).

### Genome assembly and annotation

Initial genome exploration was performed using Jellyfish v2.2.10 (*kmer size = 21*; Marçais and Kingsford 2011) in combination with GenomeScope v2 (Ranallo-Benavidez et al. 2020) to estimate genome size and heterozygosity. High-Fidelity (HiFi) reads were assembled into contigs using hifiasm v0.19 with default settings (Cheng et al. 2021). To identify and assess potential contamination, we employed BlobTools v2 (Laetsch and Blaxter 2017), which integrates taxonomic information with GC content and read coverage. To reduce assembly redundancy, one round of Purge_dups v1.2.5 (Guan et al. 2020) was applied to remove putative haplotigs. Genome coverage was assessed by remapping PacBio HiFi reads to the assembled genome using minimap2 v.2.22 (Li 2018), and coverage statistics were calculated with samtools v1.13 (Danecek et al. 2021; Supplementary Fig. 1). Genome completeness was evaluated using BUSCO v5.2.2 (Manni et al. 2021) against the arthropoda_odb10 database. Repetitive elements were identified and soft-masked using RepeatMasker v4.1.2-p1 (Smit et al. 2015) in conjunction with a custom repeat library generated using RepeatModeler v2.0.2 (Flynn et al. 2020).

Transcriptome reads were initially processed with Cutadapt v1.2.1 (Martin 2011) to remove adapter sequences. Reads were then quality-trimmed using Sickle (available at: https://github.com/najoshi/sickle), with a minimum quality score of 20 and a minimum read length of 15 bp. Filtered reads were then used to support genome annotation with Funannotate v1.8.17 (Palmer and Stajich 2017). Within this pipeline, a genome-guided transcriptome assembly was first generated with Trinity v2.8.5 (Grabherr et al. 2011) to provide transcript evidence for training gene predictors. Funannotate then combined *ab initio* predictions with transcript and protein evidence to generate consensus gene models, which were further refined to improve gene boundaries. Functional annotation was subsequently performed with InterProScan v5.32.71.0 (Jones et al. 2014) and eggNOG-mapper v2.1.6 (Huerta-Cepas et al. 2019). Funannotate was executed with the option: –*max_intronlen 100000 –repeats2evm –organism other*.

### β-lactam gene screening and phylogenetic analysis

To investigate the presence of genes involved in the biosynthesis of β-lactam compounds, we screened high-quality annotations of Collembola species available from NCBI and other repositories (Supplementary Table 1). Reference sequences of bacterial and fungal taxa for each enzyme included in the KEGG pathway for β-lactam biosynthesis (map00311) were retrieved from UniProtKB. These reference proteins were used as queries in BLASTp similarity searches against the Collembola proteomes, applying a minimum identity threshold of 35% and query coverage of at least 80%.

For phylogenetic inference, we used the same set of high-quality Collembola proteomes described above. Species trees were reconstructed with OrthoFinder v3.1.0 (Emms and Kelly 2019), employing DIAMOND in ultra-sensitive mode (Buchfink et al. 2015).

## Results and discussion

### Assembly

**Fig. 2.**
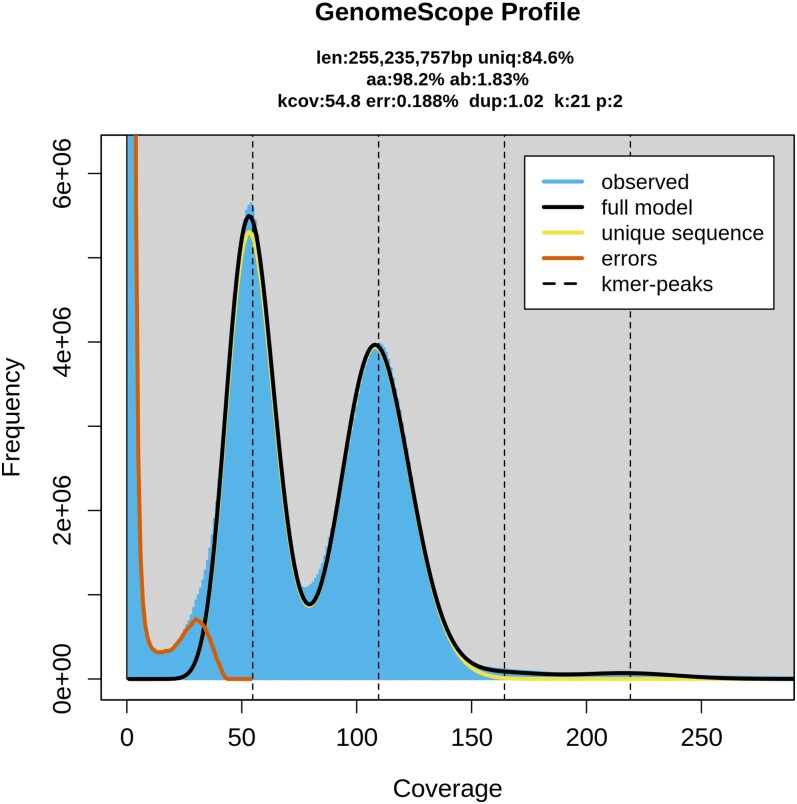
K-mer frequency distribution of raw PacBio reads generated using GenomeScope with *k* = 21. The plot displays the observed k-mer frequencies (blue area) overlaid with the GenomeScope model fit (black line).

PacBio HiFi sequencing yielded a total of 2.76 million reads, with an average read length of approximately 11 kb and a mean quality score of Q39. GenomeScope analysis estimated a haploid genome size of ∼255 Mb with a heterozygosity rate of ∼1.8% (Fig. 2). The elevated heterozygosity likely reflects the wild origin of the specimen, as it was collected directly from natural populations rather than from long-term laboratory cultures or inbred lines, as customary in other genome projects. This is further supported by the k-mer profile, where the heterozygous peak at ∼55 × coverage occurs at approximately half the depth of the heterozygous peak at ∼110 × coverage (Fig. 2).

**Fig. 3.**
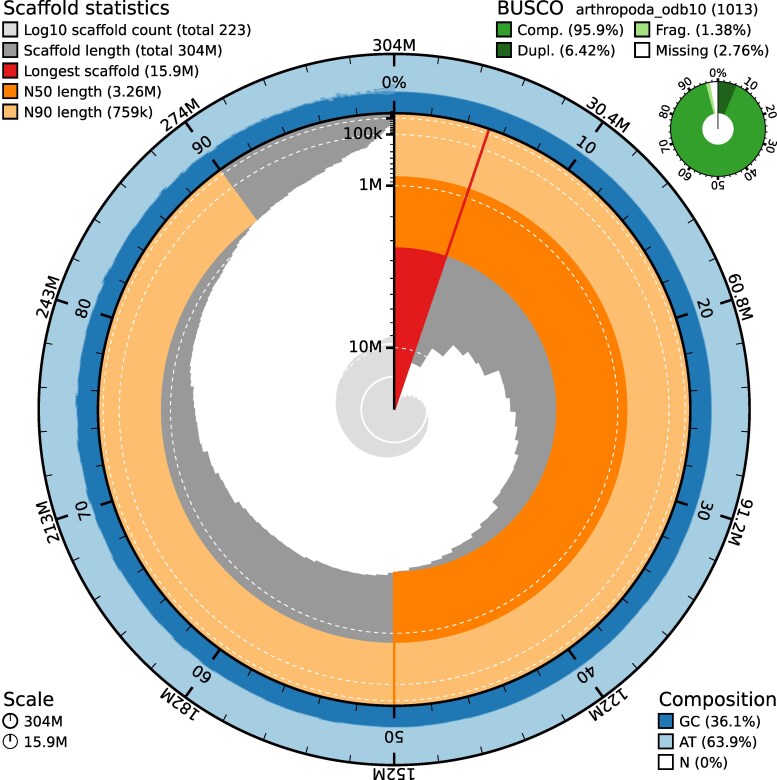
BlobTools snail plot illustrating assembly statistics, including total assembly length, N50 and N90 values, GC content, BUSCO completeness (calculated using the arthropoda_odb10 lineage), and the total number of contigs.

**Fig. 4.**
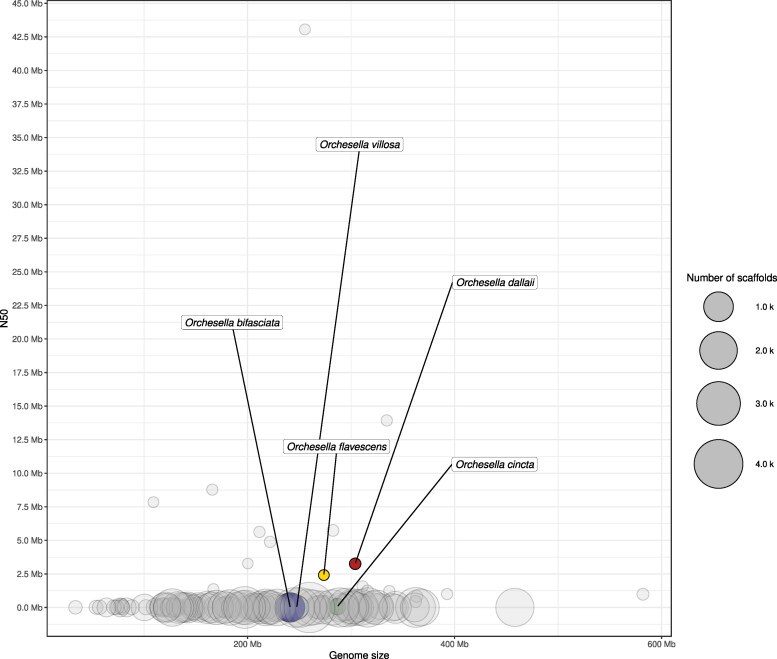
Bubble plot representing assembly metrics of all available genomes of springtails (*n* = 134) as of April 2025. Genome size and contiguity (N50) are shown on the *x*- and *y*-axes, respectively, while bubble size corresponds to the number of scaffolds—the smaller the bubble, the better the assembly continuity. *Orchesella* species are color-coded to facilitate comparison with the newly sequenced *O. dallaii* genome, which is highlighted in red.

The genome of *O. dallaii* was initially assembled into 352 primary contigs totaling 327 Mb, with an N50 of 3.02 Mb. Following the removal of potential haplotigs, the final assembly consisted of 223 contigs covering 304 Mb and exhibited an improved N50 of 3.26 Mb (Fig. 3). The assembled genome size exceeded the GenomeScope estimate (∼304 vs ∼255 Mb), likely reflecting residual heterozygosity, repetitive elements, and alternative haplotypes that are difficult to fully collapse during assembly—an outcome also described in *Sinella curviseta* (Zhang et al. 2019). Among Collembola genomes, the *O. dallaii* assembly ranks among the highest-quality genomes, positioned 10th out of 134 based on N50, and, contig-wise, is the most contiguous assembly reported for congeneric *Orchesella* species (Fig. 4). BUSCO analysis revealed high genome completeness (95.9%) alongside low duplication of single-copy orthologs (6.42%), indicating low levels of uncollapsed haplotypes (Fig. 3). Although lower duplication levels are often reported in Collembola genomes (e.g. 4.4% in the phylogenetically related *O. cincta*), higher values have also been documented, such as in *Holacanthella duospinosa* (8.0%; Zhang et al. 2019) and *Orchesella flavescens* (7.6%; McCulloch et al. 2025). Thus, the duplication rate observed in *O. dallaii* (∼6.4%) falls within the variability already known for the group.

To further validate assembly quality and rule out the possibility of residual contamination, we applied BlobTools, which did not detect contigs of clear non-metazoan origin (Supplementary Fig. 2). A small fraction of contigs (11/223), accounting for 2.3% of the total assembly length, were initially assigned to non-arthropod phyla, but a manual revision with megablast revealed significant matches to Arthropoda for all but 3 short contigs (0.1% of the total assembly length) with only weak similarity (Supplementary Fig. 3). These sequences also showed GC content and coverage profiles consistent with the rest of the assembly, suggesting that the initial assignments reflected pipeline misclassifications rather than genuine contamination. Importantly, removing these contigs led to a marked reduction in BUSCO completeness (S: 88.1%, D: 6.4%, F: 1.5%, M: 4.0%), supporting their inclusion in the final assembly. Finally, to further validate assembly completeness, we remapped PacBio HiFi long reads to the final assembly, which showed an average per-contig coverage of ∼86.2 ± 69.3, with particularly high variability in short contigs (Supplementary Fig. 1). This prevented us from confidently identifying scaffolds with exactly half coverage and thus from unambiguously assigning the sex of the sequenced specimen.

### Repetitive DNA

Although different methods were used to estimate the abundance of interspersed elements compared to the approach by Sproul et al. (2023) and Jin et al. (2024) for other springtail species, the relative abundance of transposable element (TE) classes in *O. dallaii* was grossly similar to that observed in *Yoshiicerus persimilis* (20.34%), *Tomocerus qinae* (26.11%; Jin et al. 2024), and 2 out of the 3 available genomes of *F. candida* (22.29 and 22.61%; Sproul et al. 2023; Jin et al. 2024). In contrast, TE content in *O. dallaii* was considerably lower than *H. duospinosa* (43.86%; Sproul et al. 2023) and higher than the majority of springtails analyzed by previous researchers (see Sproul et al. (2023) for a better overview). The lack of a phylogenetic pattern suggests that these differences likely reflect species-specific variation rather than an evolutionary trend of more general significance. Additionally, a substantial proportion of TEs in *O. dallaii* remained unclassified, complicating downstream interpretation (Table 1).

**Table 1.** Repetitive DNA content in the *Orchesella dallaii* genome.

	Number of elements	Sequence (%)
Retroelements	9,769	2.76
Penelope	1,103	0.15
LINEs	4,963	1.07
CRE/SLACS	193	0.03
L2/CR1/Rex	1,627	0.38
R1/LOA/Jockey	13	0
R2/R4/NeSL	68	0.02
RTE/Bov-B	843	0.29
L1/CIN4	25	0.01
LTR elements	4,806	1.69
BEL/Pao	2,245	0.75
Ty1/Copia	341	0.07
Gypsy/DIRS1	2,138	0.83
Retroviral	82	0.03
DNA transposons	3,819	0.72
hobo-Activator	1,406	0.23
Tc1-IS630-Pogo	312	0.07
Other (Mirage, *P*-element, Transib)	90	0.03
Rolling-circles	5,570	0.57
Unclassified	143,336	16.69
Total interspersed repeats		20.17

### Gene annotation

The annotation process yielded 31,769 genes, represented by 34,084 transcripts. BUSCO assessment using the Arthropoda dataset confirmed a high level of completeness (Supplementary Fig. 4). At the gene annotation level, 96.7% of BUSCOs were complete, with 90.3% single-copy and 6.6% duplicated, while only 1.3% were fragmented and 1.8% missing, in line with BUSCO values calculated at the assembly level. When transcript isoforms were also considered, the proportion of duplicated BUSCOs increased to 12.2%, reflecting the expected inflation caused by alternative splicing. This distinction highlights that the underlying gene annotation is consistent with other high-quality Collembola genomes, while the transcript-level BUSCO primarily captures isoform diversity (Cucini et al. 2024).

### β-lactam biosynthetic pathway

**Fig. 5.**
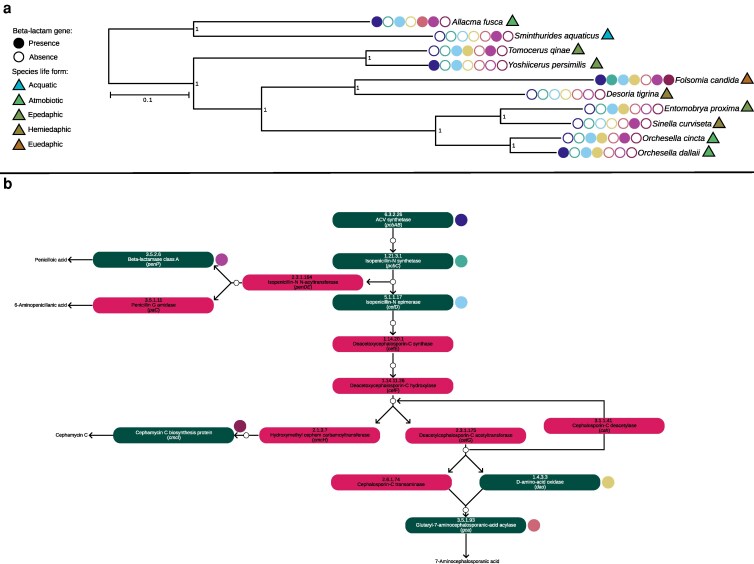
a) Phylogenetic distribution of β-lactam biosynthesis genes across selected Collembola species. The phylogeny was inferred from OrthoFinder orthologs and depicts relationships among species. Ecological life forms (aquatic, atmobiotic, epedaphic, hemiedaphic, and euedaphic) are indicated by filled triangles; life-form assignments follow Potapov et al. (2016) and original species description when available (e.g. Yu et al. 2016, 2017). Circles next to each tip show the presence (filled) or absence (empty) of β-lactam biosynthesis genes, with colors corresponding to specific enzymes as shown in panel b. Bootstrap support values are reported on branches. b) β-lactam biosynthesis pathway (KEGG map00311). Each box represents an enzyme, annotated with its EC number, enzyme name, and gene name. Green boxes indicate enzymes for which orthologs were detected in the surveyed Collembola, red boxes indicate absence. Empty circles represent reaction products; filled, colored circles adjacent to boxes map the gene symbols shown in panel a. See Methods for orthology criteria and detection thresholds.

The OrthoFinder-based phylogeny is broadly concordant with the recent reconstruction proposed by Yu et al. (2024), with strong bootstrap support across all nodes (Fig. 5). Mapping β-lactam biosynthesis genes onto this tree revealed that, with the notable exception of *Desoria tigrina*, all surveyed springtails possess at least 1 detectable ortholog associated with the β-lactam synthesis pathway under the orthology and similarity thresholds applied in this study (see Materials and Methods). However, none of the species examined harbors the complete set of enzymes required to reconstruct a complete *de novo* β-lactam biosynthetic pathway from genomic data alone, a pattern already suggested by Suring et al. (2017). Among the taxa analyzed, *F. candida* shows the most complete gene set, carrying key enzymes such as ACVS and IPNS (Roelofs et al. 2013; Suring et al. 2017), whereas all other species lack 1 or both of these core genes (Fig. 5). Our findings, therefore, partly confirm earlier studies, corroborating the unique role of *F. candida* in harboring the most extensive β-lactam complement, but also refine them by showing that other species do not follow the same presence/absence patterns previously reported, likely due to differences in data type and detection criteria.

Overall, the distribution of β-lactam biosynthesis genes does not follow a clear phylogenetic or ecological pattern (Fig. 5). Although earlier studies reported an association between β-lactam gene presence and a euedaphic (soil-dwelling) lifestyle, our genome annotation-based survey only partially supports this pattern. Among the species analyzed, *F. candida*—the sole true euedaphic taxon in our dataset—retains the most complete complement of β-lactam genes, consistent with previous reports. However, other species with different ecological strategies also harbor partial subsets of pathway genes, indicating that while soil-dwelling may favor retention of a broader repertoire, the distribution of β-lactam genes across Collembola cannot be explained by ecology alone. This discrepancy likely reflects a combination of factors: (i) differences in data type, as Suring et al. (2017) relied on transcriptomic evidence for species lacking genome assemblies, whereas here we systematically screened genome annotations; (ii) variation in orthology detection thresholds, since previous work retrieved only partial β-lactam genes under unspecified similarity cutoffs, while in this study we screened the entire pathway under stringent and reproducible parameters (see Materials and Methods); and (iii) limited taxon sampling, due to the absence of available annotation-level assemblies which constrains broader inferences. Furthermore, low-similarity hits were detected in a few taxa (e.g. *Sminthurides aquaticus, D. tigrina*), but these fell below our conservative thresholds and were excluded, underscoring the challenges of confidently classifying highly diverged HGT-derived sequences.

Taken together, these results are consistent with multiple, non-mutually exclusive evolutionary scenarios: a single ancient horizontal gene transfer event followed by lineage-specific retention and loss, or several independent acquisitions followed by divergence. Given the incomplete and heterogeneous distribution of β-lactam genes across species and the currently limited availability of annotated Collembola genomes, we refrain from proposing a definitive model at this stage.

### Mitochondrial structure

**Fig. 6.**
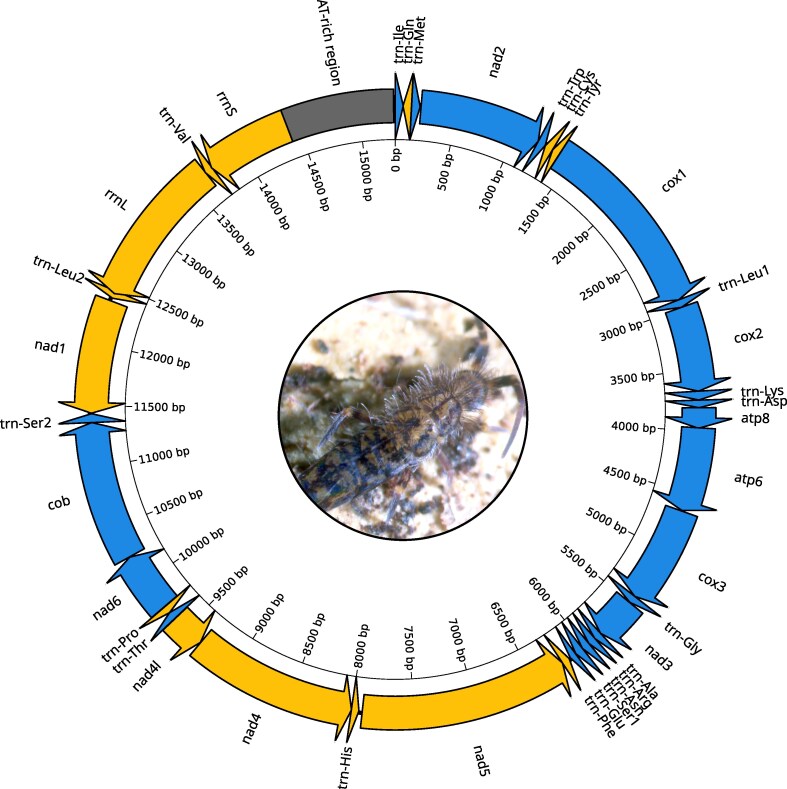
Mitochondrial genome map of *Orchesella dallaii.* Genes are represented as arrows, with blue and yellow indicating genes encoded on the J-strand and N-strand, respectively. The non-coding AT-rich region is gray colored.

The mitochondrial genome of *O. dallaii* is a circular molecule of 15,296 bp, with coverage ranging from ∼775× to 1,400× (Supplementary Fig. 5). The mitogenome encodes the typical set of 37 metazoan mitochondrial genes—13 protein-coding genes (PCGs), 22 tRNAs, and 2 rRNAs—along with a non-coding AT-rich region (Fig. 6; Supplementary Table 2). The overall nucleotide composition is strongly biased toward adenine and thymine (Supplementary Table 2). The gene order conforms to the Pancrustacea model, as commonly observed in springtails of the Entomobryomorpha order (Cucini et al. 2021b).

## Conclusion

In this study, we present the high-quality genome assembly of *O. dallaii*, the first genome sequenced from an Italian endemic springtail. Using PacBio HiFi technology combined with an ultra-low input library preparation, we achieved an assembly with excellent contiguity, ranking it among the top 10 most contiguous Collembola genomes currently available.

Beyond assembly, we performed a comprehensive annotation of both repetitive elements and PCGs, leveraging a complementary RNA-seq dataset generated from short-read sequencing. This integrative approach provides a robust and functionally informative genome annotation.

Our work provides a valuable genetic resource for a taxonomic group frequently underestimated in both biodiversity and ecological significance. By making this resource publicly available, we aim to facilitate future research in evolutionary biology, comparative genomics, and conservation genetics of springtails and other soil microarthropods. Furthermore, this genome assembly established a foundation for integrative studies that can inform conservation efforts targeting cryptic and ecologically important taxa within Mediterranean ecosystems.

## Supplementary Material

jkaf240_Supplementary_Data

## Data Availability

All data associated with this genome project have been deposited in NCBI under BioProject accession PRJEB76228. Raw sequencing reads are available in the Sequence Read Archive under accessions ERX12617126 and ERX12617127. The final genome assembly is available in NCBI under accession GCA_964186735.1, and the mitochondrial genome is available in GenBank under accession BK074881. In addition, all datasets, annotations, and assemblies employed in this study are accessible through our Figshare repository: https://doi.org/10.6084/m9.figshare.30051652. Supplemental material available at G3 online.
