# Supplementary material for: De novo genome assembly of the endemic Italian springtail Orchesella dallaii (Collembola: Orchesellidae)
Source: G3 (Bethesda). 2025 Oct 7;15(12):jkaf240. doi: 10.1093/g3journal/jkaf240 (PMC12693562; doi:10.1093/g3journal/jkaf240)
Supplement: jkaf240_Supplementary_Data [file jkaf240_supplementary_data.zip › Figure_S1_G3-2025-406193.pdf]

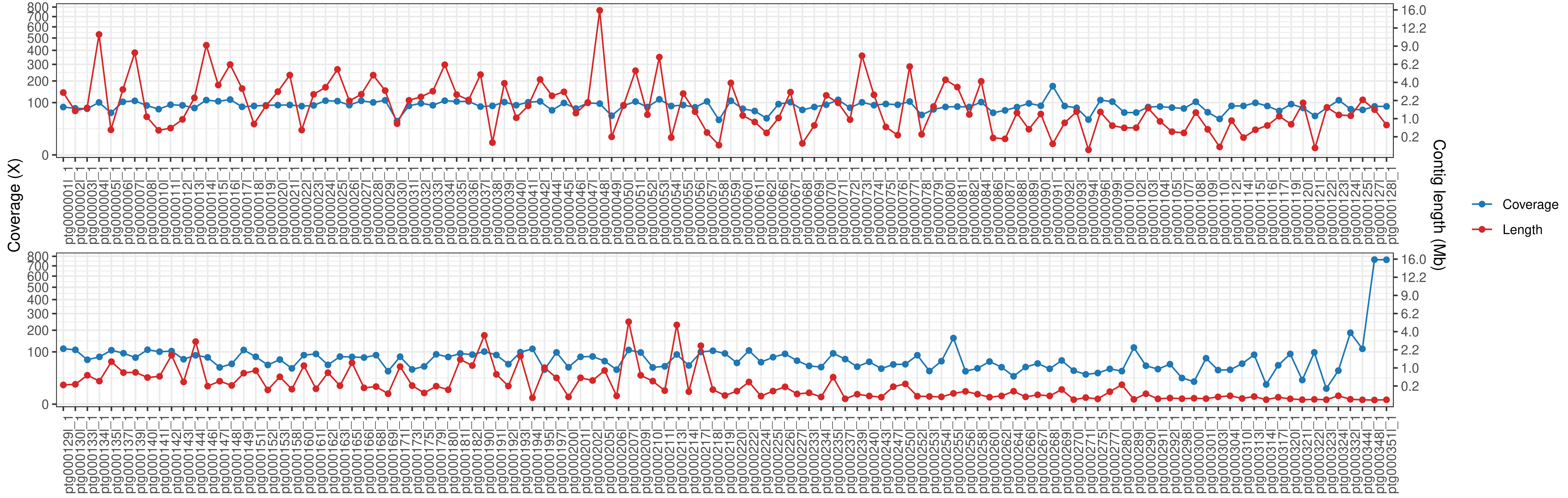

Supplementary Figure S1. Coverage and length plot of *Orchesella dallaii* contigs obained by remapping PacBio HiFi long reads over the final assembled genome and tabulated with samtools coverage. The x-axis represents individual genome contigs, while the y-axes indicate sequencing coverage and contig length.
