# Supplementary material for: De novo genome assembly of the endemic Italian springtail Orchesella dallaii (Collembola: Orchesellidae)
Source: G3 (Bethesda). 2025 Oct 7;15(12):jkaf240. doi: 10.1093/g3journal/jkaf240 (PMC12693562; doi:10.1093/g3journal/jkaf240)
Supplement: jkaf240_Supplementary_Data [file jkaf240_supplementary_data.zip › Figure_S3_G3-2025-406193.pdf]

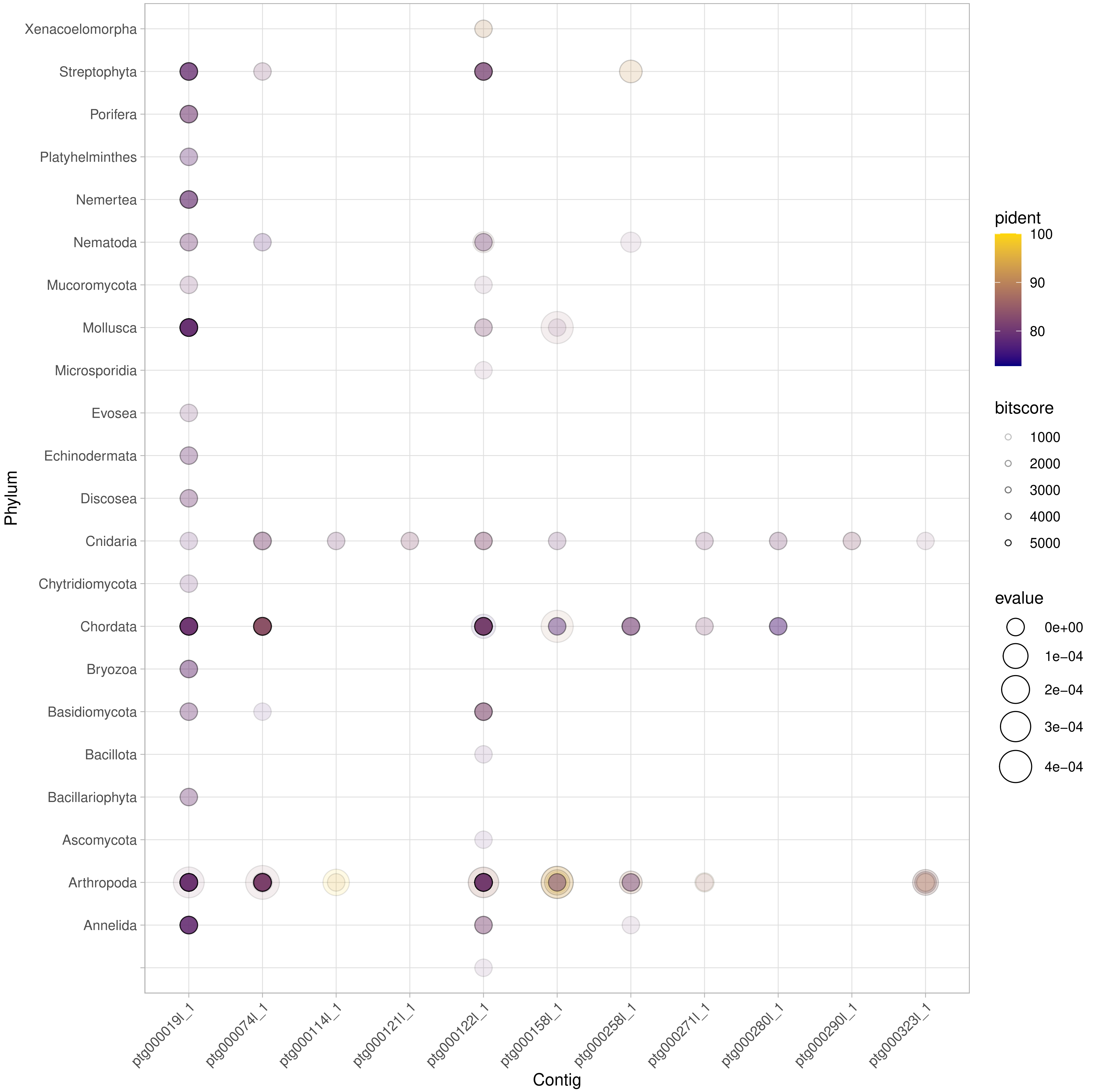


**Supplementary Figure S3.** Taxonomic classification of contaminated contigs identified by BLAST/BlobToolKit analysis after manual refinement using MEGABLAST. The plot displays tagged contaminated contigs (x-axis) across different phylogenetic groups (y-axis). Circle size represents E-value significance, with smaller circles indicating more significant matches (lower E-values). Color intensity corresponds to percent identity (pident), ranging from 80-100%, with darker colors representing higher sequence similarity. Circle transparency is proportional to bitscore values (1000-5000), where more opaque circles indicate higher alignment scores. The analysis reveals that identified contaminations screened by BlobTools/BLAST are indeed associated with multiple hits that fall within the Arthropoda lineage.
