# Supplementary material for: De novo genome assembly of the endemic Italian springtail Orchesella dallaii (Collembola: Orchesellidae)
Source: G3 (Bethesda). 2025 Oct 7;15(12):jkaf240. doi: 10.1093/g3journal/jkaf240 (PMC12693562; doi:10.1093/g3journal/jkaf240)
Supplement: jkaf240_Supplementary_Data [file jkaf240_supplementary_data.zip › Figure_S4_G3-2025-406193.pdf]

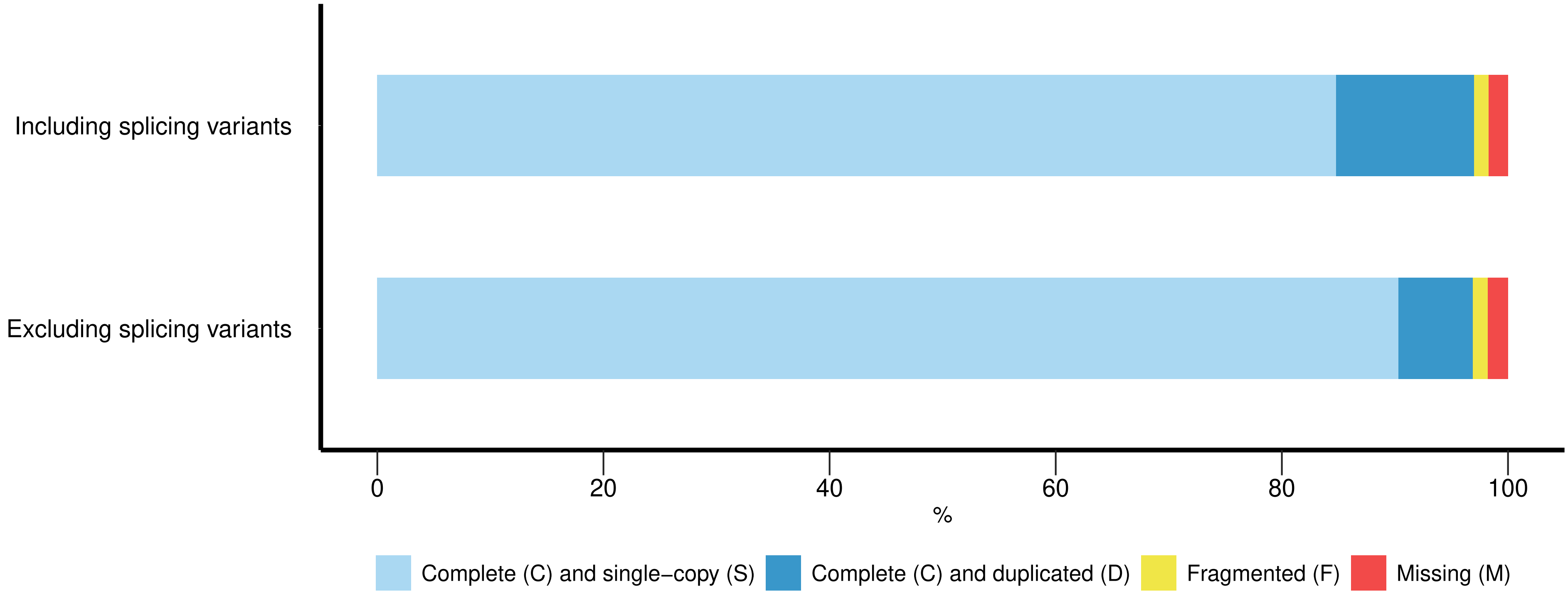

Supplementary Figure S4. BUSCO analysis through the Arthropoda lineage on the genome of *Orchesella dallaii* at the annotation level. The annotation completeness was evaluated on the full set of annotations (including splice variants) and on single-genes only.
